# Synthetic organotelluride compounds induce the reversal of Pdr5p mediated fluconazole resistance in *Saccharomyces cerevisiae*

**DOI:** 10.1186/s12866-014-0201-y

**Published:** 2014-07-26

**Authors:** Leandro Figueira Reis de Sá, Fabiano Travanca Toledo, Bruno Artur de Sousa, Augusto César Gonçalves, Ana Claudia Tessis, Edison P Wendler, João V Comasseto, Alcindo A Dos Santos, Antonio Ferreira-Pereira

**Affiliations:** 1 Instituto de Microbiologia Paulo de Góes, Departamento de Microbiologia Geral, Laboratório de Bioquímica Microbiana, CCS, Universidade Federal do Rio de Janeiro, Rio de Janeiro/RJ, Brazil; 2 Instituto de Química, Departamento de Química Fundamental, Universidade de São Paulo, Av. Prof. Lineu Prestes, 748 – building 5, Butanta, São Paulo/SP, Brazil; 3 Instituto Federal de Educação, Ciência e Tecnologia do Rio de Janeiro (IFRJ), Rio de Janeiro/RJ, Brazil; 4 Instituto de Ciências Ambientais, Químicas e Farmacêuticas, Universidade Federal de São Paulo (UNIFESP), São Paulo/SP, Brazil

**Keywords:** Organotellurides, Pdr5p, Fluconazole resistance, Saccharomyces cerevisiae

## Abstract

**Background:**

Resistance to fluconazole, a commonly used azole antifungal, is a challenge for the treatment of fungal infections. Resistance can be mediated by overexpression of ABC transporters, which promote drug efflux that requires ATP hydrolysis. The Pdr5p ABC transporter of *Saccharomyces cerevisiae* is a well-known model used to study this mechanism of antifungal resistance. The present study investigated the effects of 13 synthetic compounds on Pdr5p.

**Results:**

Among the tested compounds, four contained a tellurium-butane group and shared structural similarities that were absent in the other tested compounds: a lateral hydrocarbon chain and an amide group. These four compounds were capable of inhibiting Pdr5p ATPase activity by more than 90%, they demonstrated IC_50_ values less than 2 μM and had an uncompetitive pattern of Pdr5p ATPase activity inhibition. These organotellurides did not demonstrate cytotoxicity against human erythrocytes or *S. cerevisiae* mutant strains (a strain that overexpress Pdr5p and a null mutant strain) even in concentrations above 100 μM. When tested at 100 μM, they could reverse the fluconazole resistance expressed by both the *S. cerevisiae* mutant strain that overexpress Pdr5p and a clinical isolate of *Candida albicans*.

**Conclusions:**

We have identified four organotellurides that are promising candidates for the reversal of drug resistance mediated by drug efflux pumps. These molecules will act as scaffolds for the development of more efficient and effective efflux pump inhibitors that can be used in combination therapy with available antifungals.

## Background

The last decades have seen an increase in the immunocompromised population for several reasons including as a result of treatment of malignant diseases, HIV infection, as well as advances in organ transplantation procedures. In this scenario opportunistic infections, especially those caused by fungi, have become a serious public health problem [11]–[3]. Candidiasis is the most common fungal infection among patients with a condition that leads to immunosuppression [4],[5].

Azoles, especially fluconazole, have been commonly used to treat fungal infections [6]. However, overexpression of membrane efflux pumps by fungal cells is an important mechanism that causes azole resistance [7]. Some of these efflux pumps belong to the Pleiotropic Drug Resistance (PDR) sub-family of ATP-Binding Cassette (ABC) transporters, and they lead to active transport of drugs using energy derived from ATP hydrolysis [8].

*Saccharomyces cerevisiae* can express several ABC transporters, and of these, Pdr5p has been the best studied [9]. This efflux pump causes the extrusion of several drugs that are used to treat fungal infections. Also, it exhibits a profile of substrates and inhibitors that is similar to those of other ABC transporters that are expressed by pathogenic fungi [10]. These features make Pdrp5 a good experimental model for the study of antifungal resistance mediated by ABC transporters.

One strategy for overcoming drug resistance mediated by efflux pumps is the use of compounds that can function as chemosensitizers. These compounds potentiate the efficacy of existing azoles, such as fluconazole, by inhibiting these ABC transporters [11]. Thus, the development of novel azole chemosensitizers that increase the potency of these drugs against both sensitive and resistant fungi may allow the use of previously ineffective antifungal to treat fungal infections [12]. Some studies have already reported compounds that are capable of reversing the resistance phenotype, such as D-Octapeptides [12], enniatin [13], isonitrile [14] and gallic acid derivatives [15].

Recently, interest in organic compounds containing tellurium (Te) or selenium (Se) has increased and several studies have been published demonstrating biological properties for both elements. Despite the relative toxicity conferred by organic compounds containing tellurium [16], some studies have shown that these molecules may have immunomodulatory and anti-inflammatory properties [17], antioxidant abilities [18], and anti-proliferative actions against certain tissues [19]. Selenium is a nutritionally essential trace element for mammals. Studies have shown that some organic compounds derived from this chalcogenide exhibit antinociceptive, hepatoprotective, neuroprotective, anti-inflammatory and anti-carcinogenic properties [20]. Furthermore, some organochalcogenides containing Te or Se are capable of inhibiting the ATPase activity of the Na^+^/K^+^ ATPase that is present in rat brains [21] and can inhibit the ATPase activity of P-Glycoprotein and vinblastine efflux mediated by this neoplasic cell multidrug transporter [22]. Finally, Te and Se containing compounds can inhibit the plasma membrane H^+^-ATPase from *S. cerevisiae*[23].

Although several biological properties have already been described in the literature for chalcogenides and their derivatives, molecules containing selenium or tellurium with the capacity to reverse efflux pump-mediated azole resistance have not yet been reported. We were interested in studying the effects of organic compounds containing tellurium or selenium on Pdr5p, which is a well-known experimental model for the study of fungal resistance mediated by efflux pumps. In this study, we evaluated 13 synthetic compounds; some of which contained tellurium (Te) or selenium (Se), and others that were devoid of both chalcogenides.

## Methods

### Chemicals

Reagents were purchased from Sigma-USA (ATP-Sodium) or Tecoland-USA (FK 506-tacrolimus) unless otherwise stated. All reagents purchased were of highest available standard.

### Synthetic compounds used in this study

**Figure 1:**
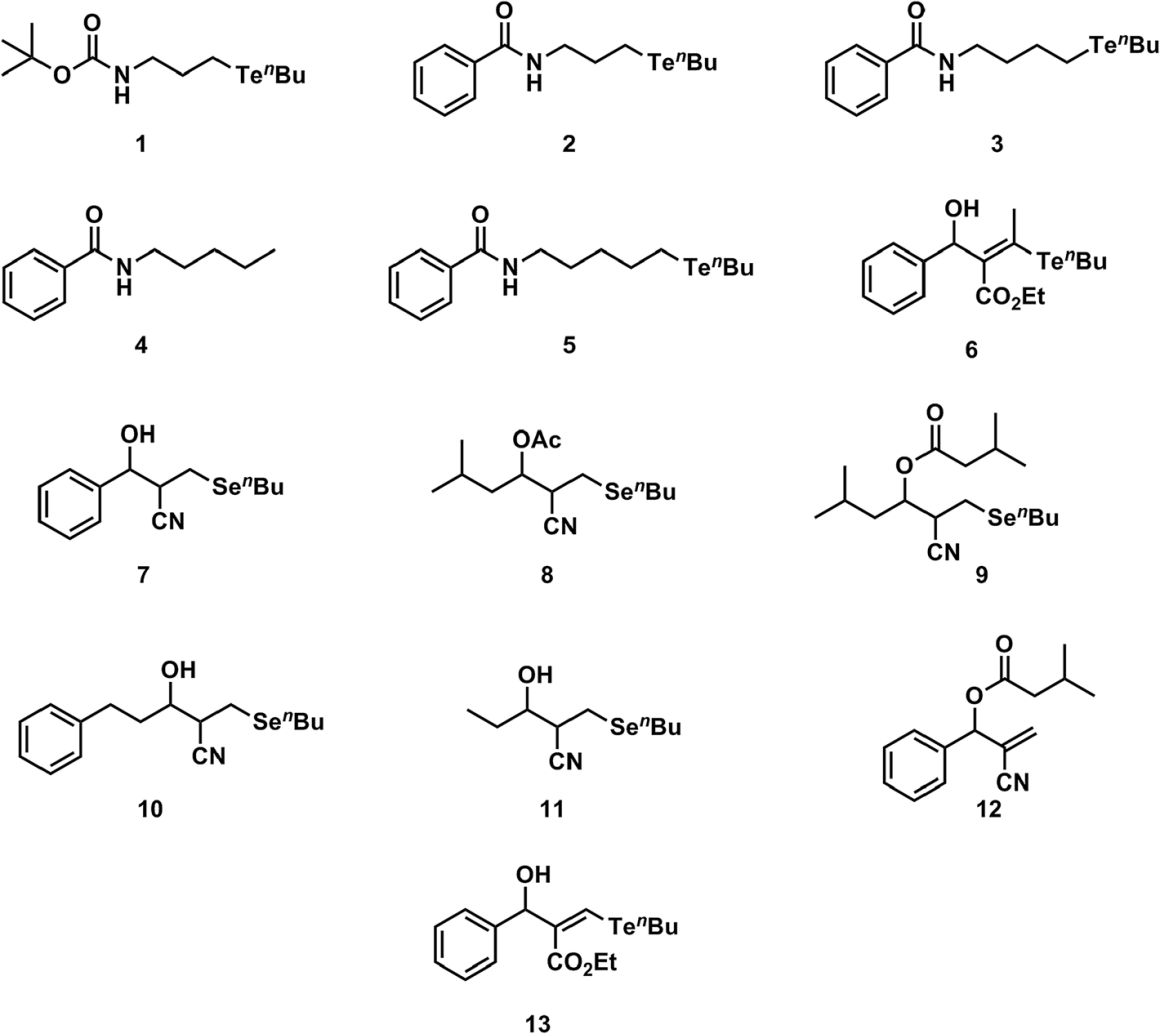
Chemical structures of the synthetic compounds studied.

The compounds listed in Figure 1 were synthesized according to procedures that had been previously developed by our group; synthetic and spectroscopic information about these compounds can be found in the original publications [24]–[27]. All of the compounds were kept in a desiccator at 4°C, and the stock solutions were prepared using dimethyl sulfoxide (DMSO) as a solvent.

### Strains and culture conditions

In this study, two mutant strains of *Saccharomyces cerevisiae* were used. The first strain AD124567 (Pdr5p+) overexpresses Pdr5p, while the genes encoding the Pdr3p regulator and the other five ABC transporters (Yor1p, Snq2p, Pdr10p, Pdr11p and Ycf1p) have been deleted. The second one AD1234567 (Pdr5p-) contains deletions of the same six genes, as well as the gene that encodes the Pdr5p transporter [28]. The yeast strains were grown in YPD medium (2% glucose, 1% yeast extract, 2% peptone) at 30°C with agitation and were harvested in the exponential phase of growth. One fluconazole resistant strain of *Candida albicans*, isolated from urine sample, was also used (approved by Instituto de Estudos em Saúde Coletiva – **IESC/UFRJ** – Protocol N° 030/2001). In this case, the yeast were cultivated in Sabouraud medium (4% glucose and 1% peptone), at 37°C under agitation (150 rpm).

### Preparation of plasma membranes

Yeast plasma membrane isolates from the *S. cerevisiae* mutant strain Pdrp5+ and from the null mutant Pdr5p- were obtained as previously described by Rangel et al. [15]. The plasma membrane preparations were stored in liquid nitrogen and thawed immediately prior to use in the Pdr5p ATPase activity assays.

### ATPase activity assay

The effect of the compounds on the ATPase activity of Pdr5p was quantified by incubating Pdr5p-containing membranes (0.013 mg/mL final concentration) in a 96-well plate at 37°C for 60 min in a reaction medium containing 100 mM Tris–HCl (pH 7.5), 4 mM MgCl_2_, 75 mM KNO_3_, 7.5 mM NaN_3_, 0.3 mM ammonium molybdate and 3 mM ATP in the presence of the synthetic compounds. After incubation, the reaction was stopped by the addition of 1% SDS, as described previously by Dulley [29]. The amount of released inorganic phosphate (Pi) was measured as previously described by Fiske & Subbarrow [30]. Preparations containing plasma membranes obtained from the null mutant strain AD1234567 (Pdr5p- membranes) were used as controls. The difference between the ATPase activity of the Pdr5p + and Pdr5p- membranes represents the ATPase activity that is mediated by Pdr5p.

### Effect of compounds on the growth of *S. cerevisiae* strains

This assay was conducted according to Niimi et al. [12]. The effect of the compounds on the growth of both mutant strains of *S.cerevisiae* used in this work was determined by microdilution assays using 96-well microplates. The cells were inoculated into YPD medium at a concentration of 1 × 10^4^ cells per well and incubated at 30°C for 48 h with agitation (150 rpm) in the presence of different concentrations of the compounds. Controls were performed using DMSO at a final concentration of 1% to verify the toxicity of the solvent used to solubilize the compounds. Cell growth was determined using a microplate reader at 600 nm (Fluostar Optima, BMG Labtech, Offenburg, Germany).

### Lytic effect of compounds on human erythrocytes

This assay was conduct as described by Niimi et al. [12]. Human erythrocytes were previously washed three times and resuspended in phosphate-buffered saline (PBS-pH 7.2). Red blood cells (final density 0.5%) were then incubate in the presence of different concentrations of the synthetic compounds for 60 min at 37°C. After incubation, the cells were pelleted by centrifugation at 3,000 *g* for 5 min and aliquots of 100 μL of the supernatant were transferred to the wells of a microplate. The absorbance of the hemoglobin released from the erythrocytes was measured at 540 nm. A control of 100% hemolysis was performed incubating the cells in the presence of PBS containing 1% Triton X-100.

### Evaluation of fluconazole resistance reversion by the synthetic compounds

The “spot test” was used as a measure of growth as previously described by Rangel et al. [15]. For *S. cerevisiae* strain Pdr5p+, 5 μL samples of fivefold serially diluted yeast cultures (initially suspended to an OD of 0.1) were spotted on YPD agar in 6 well sterile polystyrene plates. They were incubated in the presence of synthetic compounds (100 μM) only or associated with fluconazole at 120 μg/mL. Controls were performed using YPD alone and YPD supplemented with: 120 μg/mL fluconazole, 120 μg/mL fluconazole + 0.5% DMSO, 120 μg/mL fluconazole + 10 μM FK506. Plates were incubated at 30°C for 48 h.

In the case of *C. albicans*, the same methodology was used, but with some adaptations: 5 μL of a five-fold serial dilution from a yeast suspension containing 6 × 10^5^ cells/mL was spotted on Sabouraud agar supplemented with the compounds at 100 μM alone or combined with fluconazole at 64 μg/mL. The incubation of the six well plates was carried at 37°C for 48 h.

### Checkerboard assay with compounds and fluconazole using *Candida* strain from clinical isolate

*Candida albicans* cells, in exponential growth phase (2.5 × 10^3^ cells/mL) were incubated in presence of different combinations of fluconazole and compound at 37°C for 48 hours in RPMI 1640 (Sigma) using 96-well plates under stirring. Cell growth was determined using a plate reader (Fluostar Optima, BMG Labtech, Germany) at a wavelength of 600 nm. The MIC value was referred to concentration capable of causing 80% growth inhibition (MIC 80). Possible synergism between fluconazole and tested compounds was determined based on the fractional inhibition concentration index (FICI). Synergic, indifferent and antagonistic interactions were defined by a FICI of <0.5, 0.5-4.0 or 4.0 respectively [31].

### Statistical analysis

All experiments were performed in triplicate. Data were presented as mean ± standard error. A probability level of 5% (p < 0.05) in Student’s t -test was considered significant.

## Results and discussion

### ATPase activity

Pdr5p is an ABC transporter and as such the inhibition of its ATPase activity could significantly affect the efflux of fluconazole and contribute to the reversal of resistance against this antifungal. Thus, a screening assay was performed to identify synthetic compounds that could promote inhibition of ATP hydrolysis catalyzed by Pdr5p (at 100 μM final concentration).

**Figure 2:**
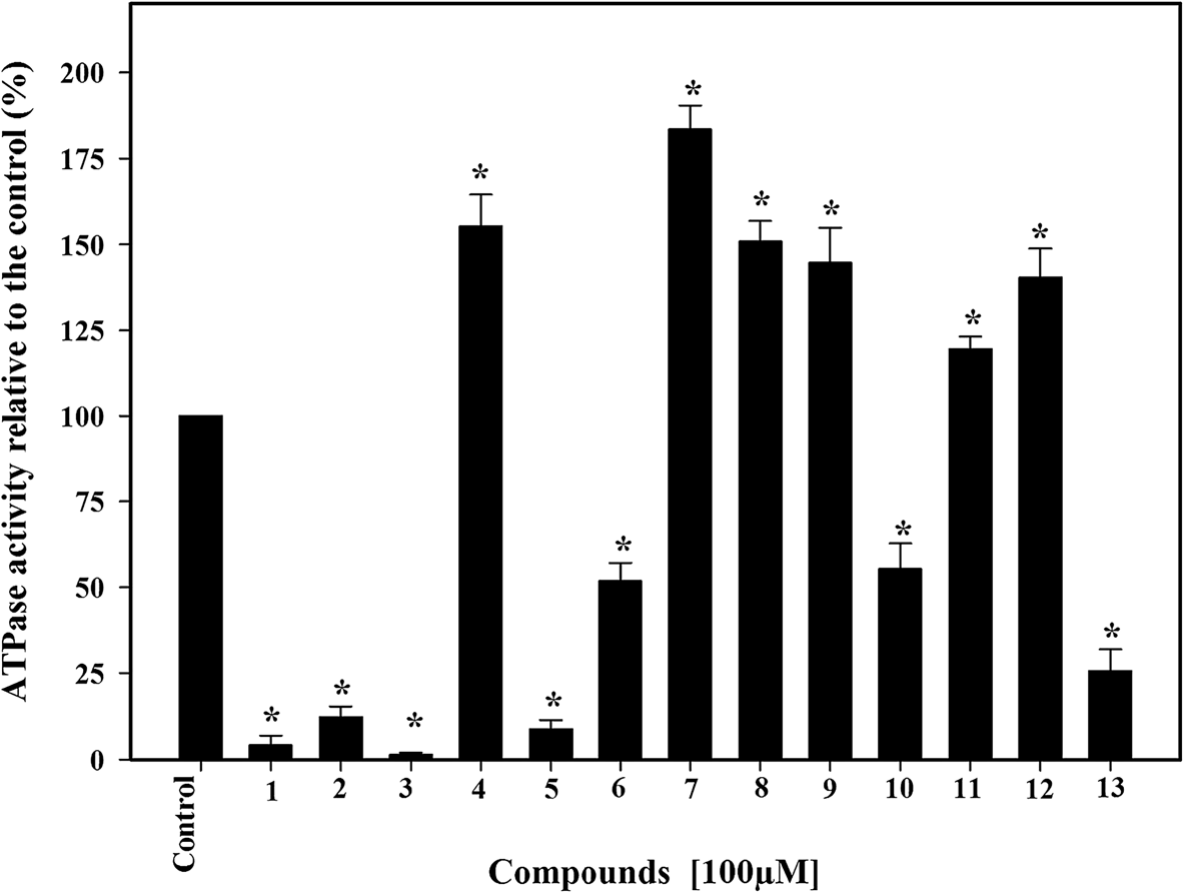
**Effect of synthetic compounds on the Pdr5p ATPase activity.** Pdr5p-enriched plasma membranes were incubated in the presence of the synthetic compounds at a concentration of 100 μM. The ATPase activity was measured as described in the Methods. The control bar represents 100% of the enzymatic activity in the absence of the compounds. The data represents means ± standard error of three independent experiments are shown, *p < 0.05.

Of the 13 compounds tested only four (1, 2, 3 and 5) were capable of inhibiting Pdr5p ATPase activity by more than 90% (Figure 2). All four compounds contained a butyl-tellurium residue, a lateral hydrocarbon chain and an amide group, that were absent in the other tested compounds. This suggests that these chemical structure could have an important role in the inhibitory process.

**Figure 3:**
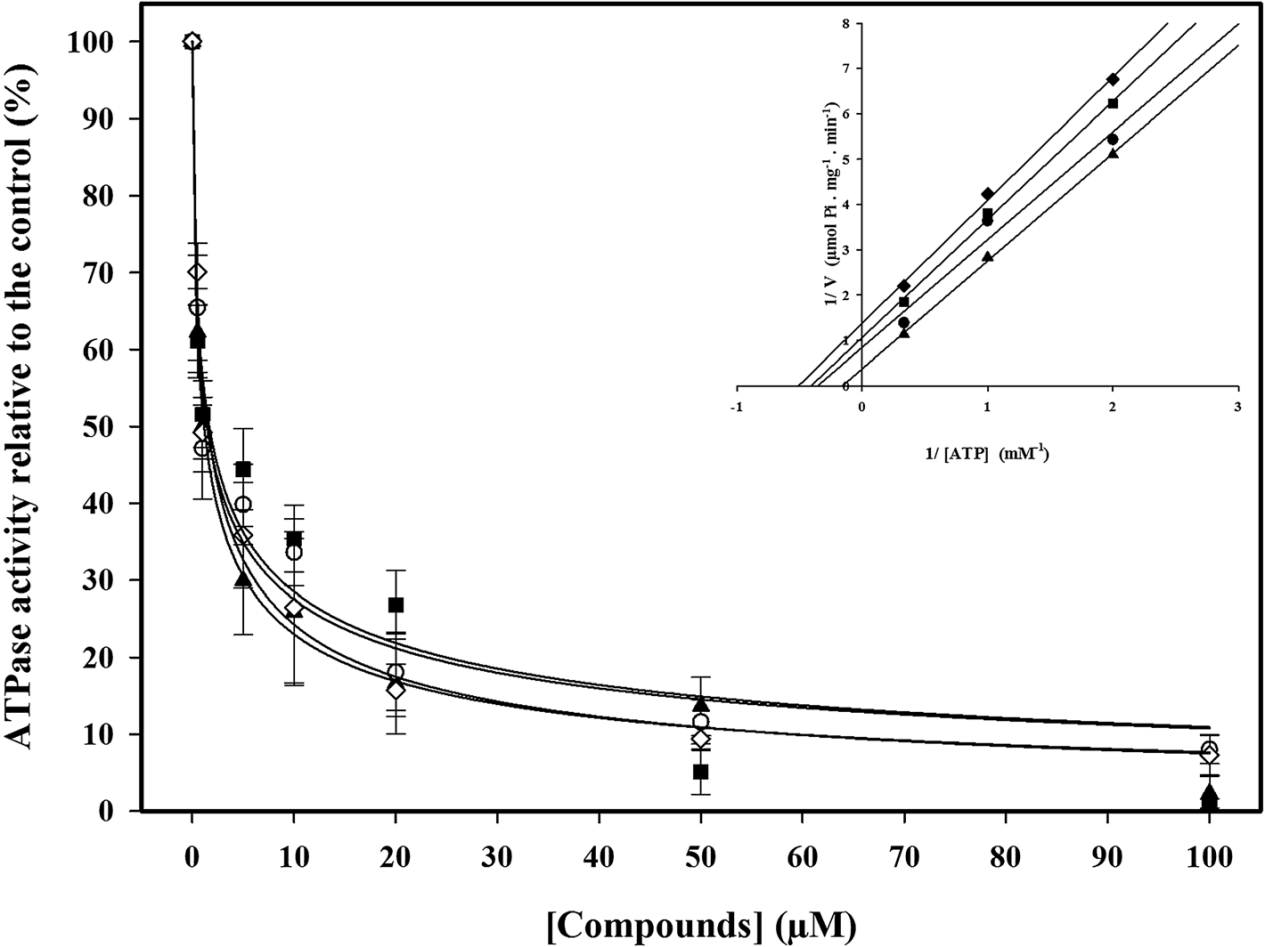
**Dose response curves of inhibition on Pdr5p ATPase activity by organotellurium compounds.** Pdr5p-enriched plasma membranes were incubated with: (▲) compound 1; (○) compound 2; (■) compound 3; (◊) compound 5. Data represent means ± SE of three independent experiments. Inset: Double reciprocal plot of compound 1: (▲) 0 μM; (●) 0.5 μM; (■) 1.0 μM; (♦) 2.0 μM. The experiment was performed using 0.5, 1 or 3 mM ATP as a substrate. The data represent means of three independent experiments.

The four active compounds (1, 2, 3 and 5) were selected for further investigation. Dose–response curves and a double reciprocal plot were performed (Figure 3). The results confirm that these compounds are strong inhibitors of Pdr5p ATPase activity and exhibit IC_50_ values lower than 2 μM (Table 1). Furthermore, the double reciprocal plot for compound 1 demonstrated an uncompetitive pattern of inhibition (Figure 3). Compounds 2, 3 and 5 demonstrated the same mechanism of inhibition (data not shown).

**Table 1:** **The IC**_**50**_**values of the compounds against the ATPase activity of Pdr5p**

**Compounds**	**IC**_**50**_**(μM)**
**1**	1.14 ± 0.21
**2**	1.45 ± 0.49
**3**	1.74 ± 0.91
**5**	1.48 ± 0.32

The data represent the means ± standard error of three independent experiments.

Until now, there have been no reports in the literature of organic synthetic compounds containing tellurium that inhibit Pdr5p ATPase activity. However, many other molecules, of synthetic or natural origin, also exhibit this ability. Silva et al. [32] demonstrated that oroidin, a derivative of a compound from a sponge, is able to inhibit the catalytic activity of this multidrug transporter with an IC_50_ of 20 μM. Rangel et al. [15], while studying gallic acid derivatives, observed that decyl gallate has an IC_50_ value of 13.5 μM. Both compounds competitively inhibit the enzyme activity of Pdr5p. Competitive inhibition is a more common characteristic than the uncompetitive inhibition shown by the four organotellurides.

As mentioned by Cannon et al. [11], inhibition of plasma membrane H^+^-ATPase activity could contribute to the reversal of ABC transporter-mediated azole resistance, by depleting the intracellular ATP concentration. To investigate this, the effects of the four organotellurides (1, 2, 3 and 5) on the plasma membrane H^+^-ATPase of *S. cerevisiae* were evaluated. The organotellurides leaded a powerful inhibition of the H^+^-ATPase activity (more than 90%) and exhibited IC_50_ values of approximately 2.7 μM (data not shown). Chan and colleagues [23] previously demonstrated that Ebselen, a well-known organoselenium compound, was also able to inhibit the activity of *S. cerevisiae* plasma membrane H^+^-ATPase in a dose dependent manner. Ebselen was also shown to be toxic for *S. cerevisiae* at a concentration of 10 μM, unlike the organotellurides investigated in this study.

### Effect of the compounds on the growth of Pdr5p+ and Pdr5p- mutant *S. cerevisiae* strains

**Figure 4:**
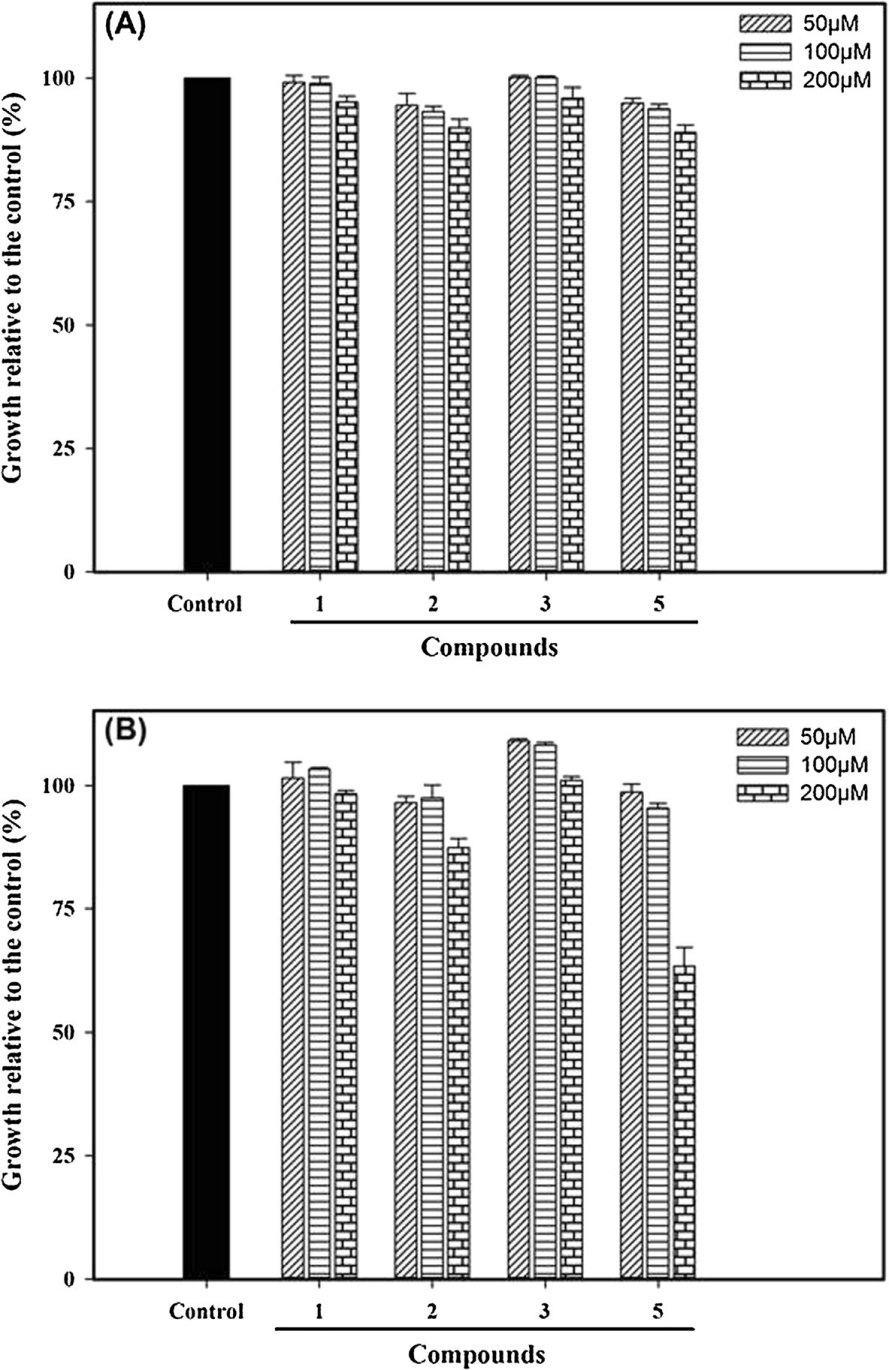
**Effect of organotellurides on the growth of*S. cerevisiae*mutant strains (A) AD124567 and (B) AD1234567.** The yeast cells were incubated in different concentrations (inset) of compounds 1, 2, 3 and 5. The control bar represents 100% of growth in the absence any compounds. The data represent the means ± standard error of three independent experiments.

The organotellurides 1, 2, 3 and 5 that inhibited Pdr5p activity did not affect the growth of the Pdr5p+ strain at concentrations up to 200 μM (Figure 4A). However, the growth of the Pdr5p- null mutant was significantly impaired after incubation with 200 μM of compound 5 (Figure 4B). This indicates that this compound could work as substrate and ATPase activity inhibitor of Pdr5p such as FK506, a classical and potent Pdr5p inhibitor [33].

### Evaluation of cytotoxicity against human erythrocytes

**Figure 5:**
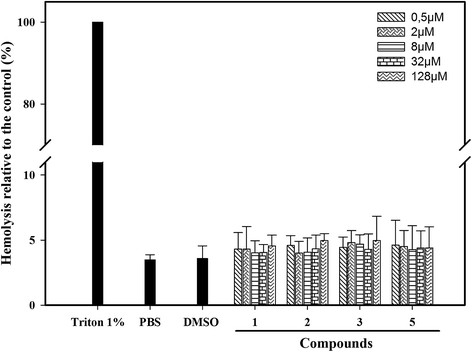
**Hemolytic activity of organotellurides on human erythrocytes.** A human erythrocyte suspension (0.5%) was incubated in the presence of compounds 1, 2, 3 and 5 at different concentrations (inset). Controls: The 100% of hemolisys – PBS with Triton 1%; DMSO control – PBS with DMSO 0.8%, and PBS control – added with no compounds. The data represent the means ± standard error of three independent experiments.

The active compounds were tested for their hemolytic activity on human erythrocytes (Figure 5). As shown in Figure 5, even at the highest concentration used in this assay (128 μM) the four compounds promoted the release of around 4% of erythrocyte hemoglobin. There was no significant difference between the hemolysis caused by the compounds and that observed in PBS (3.5% hemolysis) and DMSO (3.7% hemolysis) controls. Therefore, all four active compounds showed another a desirable feature of a compound to reverse fluconazole resistance that is a low toxicity for a mammal cell line.

### Fluconazole resistance reversion by the synthetic organotellurides

**Figure 6:**
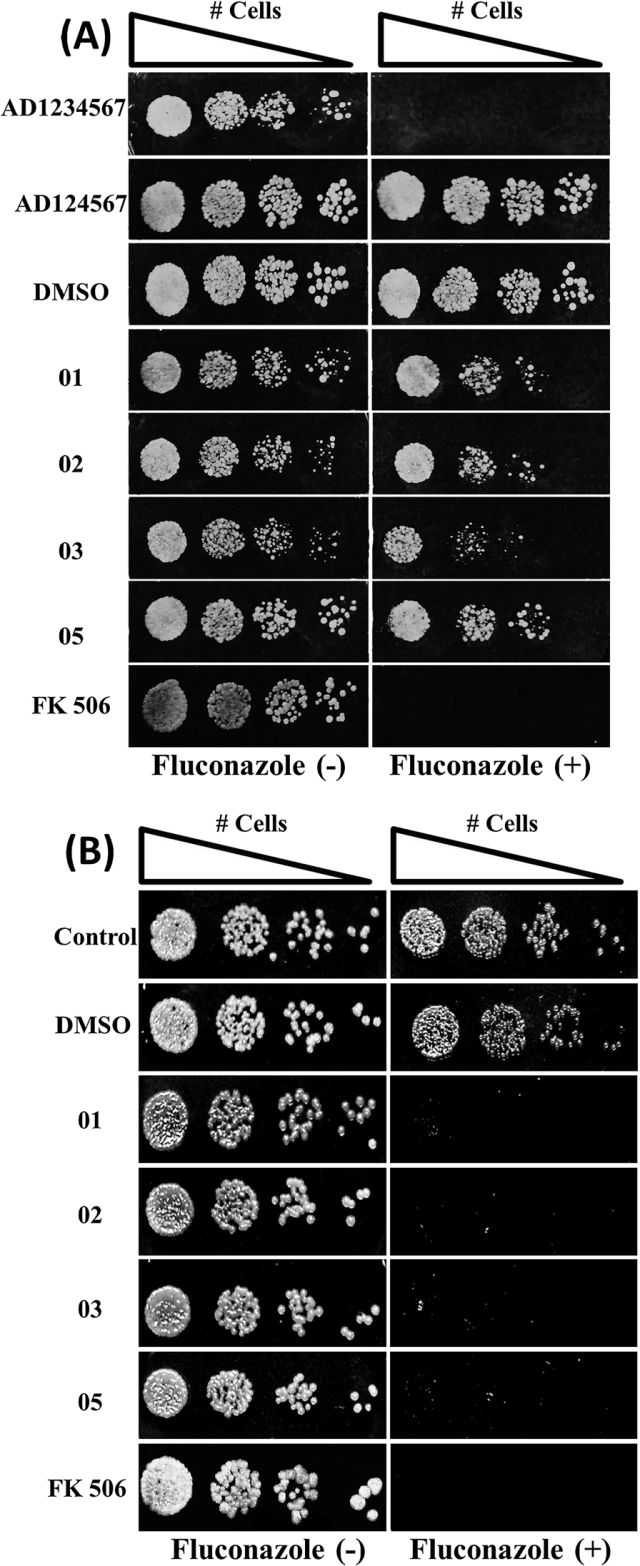
**Evaluation of the reversion of the fluconazole resistance by the organotellurides. (A)** AD124567 strain of *S. cerevisiae*: Fluconazole (−): yeast cell growth on YPD solid in absence of fluconazole. Fluconazole (+): yeast cell growth on YPD solid medium in presence of fluconazole at 120 μg/mL. Medium containing FK506 10 μM + fluconazole 120 μg/mL was used as positive control. **(B)** Resistant *Candida albicans* strain (clinical isolate): Fluconazole (−): yeast cell growth on Sabouraud solid medium in absence of fluconazole. Fluconazole (+): yeast cell growth on Sabouraud solid in presence of fluconazole at 64 μg/mL.

The spot assay shown in Figure 6A demonstrates that Pdr5p+ strain, which is resistant to fluconazole (MIC = 600 μg/mL), was able to grow on a medium containing fluconazole at 120 μg/mL as well as in presence of compounds at 100 μM. However, an evident reduction in growth was observed when this strain was incubated in presence of fluconazole (120 μg/mL) associated with any of the four organotellurides (100 μM). Thus, it was possible to demonstrate that these synthetic compounds were able to reverse the fluconazole resistance mediated by Pdr5p in a manner similar to the reversion promoted by FK506. A control using the Pdr5p- null mutant (fluconazole sensitive strain (AD1234567)) was performed to confirm that the presence of Pdr5p is responsible for the fluconazole resistance of the AD124567 strain.

The same experiment was performed using a fluconazole resistant *Candida albicans* clinical isolate because overexpression of efflux pumps is a possible mechanism of resistance to azoles in this yeast also. However, the level of expression of the *C. albicans* ABC transporter (CaCdr1p) is lower in comparison to the *S. cerevisiae* strains used in the present work that were genetically modified to overexpress the efflux pump (Pdr5p). Thus, the hypothesis is that would be possible to reverse the resistance in pathogenic yeast, as resistant *C. albicans* from a clinical isolate, with lower concentration of azole in comparison with AD124567 strain. The *C. albicans* clinical isolate was able to grow in presence of fluconazole at 64 μg/mL (Figure 6B) that is considered as resistant strain. The active compounds alone (100 μM) did not affect growth of *C. albicans*, but when associated with fluconazole (64 μg/mL) were able to promote a complete growth inhibition in comparison with inhibition obtained in the presence of FK506 (Figure 6B). This data reinforces the results obtained with *S. cerevisiae* and provides further evidence that blocking efflux pumps represents a valid therapy measure for treatment of resistant fungal strains. This strategy becomes more evident using the checkerboard assay where compounds and fluconazole were tested in different concentrations (Table 2). All compounds tested were able to act synergistically with fluconazole since they showed FICI values lower than 0.5 [31]. This proves the efficiency of the use of those organotellurides in combination with azoles in reversion of resistance due to overexpression of efflux pumps in pathogenic fungi such as *C. albicans*.

**Table 2:** Checkerboard assay* using ***Candida albicans*** **strain**

	**Compound**			**Fluconazole**				
**Compounds**	**MIC (μg/mL)**	**MIC combined (μg/mL)**	FIC*	**MIC (μg/mL)**	**MIC combined (μg/mL)**	**FIC**^**a**^	**FICI**^**b**^	**Outcome**
1	68.4	4.3	0.063	256	4	0.016	0.079	Synergy
2	70.0	4.4	0.063	256	4	0.016	0.079	Synergy
3	74.4	2.3	0.030	256	4	0.016	0.046	Synergy
5	74.9	2.3	0.031	256	4	0.016	0.047	Synergy

*This assay was done with organotellurides and fluconazole isolated or combined.

MICs were determined by a microdilution technique based on 80% reduction of growth. ^a^FIC = fractional inhibitory concentration; ^b^FICI = fractional inhibitory concentration index.

## Conclusions

Compounds 1, 2, 3 and 5 are synthetic compounds that have some similarities. Firstly, all they contain a butyl tellurium residue, secondly, they have a lateral hydrocarbon chain and finally, they all possess an amide group. All they were able to reverse the fluconazole resistance mediated by Pdr5p from *S. cerevisiae*. A likely mechanism for this reversal is the direct inhibition of the ATPase activity of Pdr5p and the indirect inhibition of the plasma membrane H^+^-ATPase.

Furthermore, the compounds could also overcome fluconazole resistance expressed by a clinical isolate of *Candida albicans.* The reversal of fluconazole resistance was obtained using 100 μM of the compounds. This concentration did not demonstrate toxicity against human erythrocytes or fungal cells. In conclusion, these compounds could be promising candidates for the reversal of resistance mediated by drug efflux pumps, act synergistically with fluconazole and could serve as prototypes for the synthesis of other molecules that could be capable of inhibiting efflux pumps with greater efficiency.

### Availability of supporting data

The data sets supporting the results of this article are included within the article.

## Competing interests

The authors declare that they have no competing interests.

## Authors’ contributions

LFRS: Carried out the conception and design the experiments; the acquisition, analysis and interpretation of data. He also drafts the manuscript. FT: Carried out the synthesis of the compounds used in this work and was involved in revising the manuscript critically. BAS: Carried out the synthesis of the compounds used in this work, and was involved in revising the manuscript critically. ACG: Carried out the synthesis of the compounds used in this work, and was involved in revising the manuscript critically. ACT: Had made contributions to analysis and interpretation of data and was involved in revising the manuscript critically. EPW: Carried out the synthesis of the compounds used in this work, and was involved in revising the manuscript critically. JVC: Carried out the supervision of the students involved in the synthesis of the compounds used in this work, and was involved in revising the manuscript critically. AAS: Designed the synthesized compounds and carried out the supervision of the students involved in the synthesis of the compounds used in this work, and was involved in revising the manuscript critically. He was involved in revising the manuscript critically and gave final approval of the final version. AFP: Helped with the conception and design the experiments; with analysis and interpretation of data and draft the manuscript. He was involved in revising the manuscript critically and gave final approval of the version to be published. All authors read and approved the final manuscript.
